# Finger burns caused by concentrated hydrofluoric acid, treated with intra-arterial calcium gluconate infusion: case report

**DOI:** 10.1590/S1516-31802009000600011

**Published:** 2010-05-21

**Authors:** Eduardo Mello De Capitani, Elcio Shiyoti Hirano, Isabela de Souza Cortez Zuim, Laura Bertanha, Ronan José Vieira, Paulo Roberto Madureira, Fábio Bucaretchi

**Affiliations:** I MD, PhD. Associate professor, Department of Internal Medicine, Poison Control Center, School of Medicine, University Hospital, Universidade Estadual de Campinas (Unicamp), Campinas, São Paulo, Brazil.; II MD. Medical doctor, Emergency Department, University Hospital, Universidade Estadual de Campinas (Unicamp), Campinas, São Paulo, Brazil.; III Undergraduate medical student, Poison Control Center, School of Medicine, University Hospital, Universidade Estadual de Campinas (Unicamp), Campinas, São Paulo, Brazil.; IV MD, PhD. Assistant professor, Department of Internal Medicine, Poison Control Center, School of Medicine, University Hospital, Universidade Estadual de Campinas (Unicamp), Campinas, São Paulo, Brazil.; V MD, PhD. Assistant professor, Department of Preventive and Social Medicine, Poison Control Center, School of Medicine, University Hospital, Universidade Estadual de Campinas (Unicamp), Campinas, São Paulo, Brazil.; VI MD, PhD. Assistant professor, Department of Pediatrics, Poison Control Center, School of Medicine, University Hospital, Universidade Estadual de Campinas (Unicamp), Campinas, São Paulo, Brazil.

**Keywords:** Hydrofluoric acid, Fingers, Calcium gluconate, Infusions, intra-arterial, Caustics, Ácido fluorídrico, Dedos, Gluconato de cálcio, Infusões intra-arteriais, Cáusticos

## Abstract

**CONTEXT:**

Hydrofluoric acid (HF) is widely used in industry and at home. Severe lesions can occur after contact with highly concentrated solutions, leading to tissue necrosis and bone destruction. Specific treatment is based on neutralization of fluoride ions with calcium or magnesium solutions.

**CASE REPORT:**

A 41-year-old male was seen at the emergency department 35 minutes after skin contact with 70% HF, showing whitened swollen lesions on the middle and fourth fingers of his right hand with severe pain starting immediately after contact. 2.5% calcium gluconate ointment was applied. Twenty-four hours later, the patient was still in severe pain and the lesions had worsened. Considering the high concentration of the solution, early start of severe pain, lesion characteristics and impossibility of administering calcium gluconate subcutaneously because of the lesion location, the radial artery was catheterized and 2% calcium gluconate was administered via infusion pump for 36 hours, until the pain subsided. No adverse effects were seen during the procedure. Ten days later, the lesions were stable, without bone abnormalities on X-rays. Six months later, a complete recovery was seen.

**CONCLUSIONS:**

Intra-arterial calcium gluconate might be considered for finger burns caused by concentrated HF. Complete recovery of wounded fingers can be achieved with this technique even if started 24 hours after the exposure. However, controlled clinical trials are needed to confirm the effectiveness and safety of this intervention.

## RESUMO

**CONTEXTO:**

Ácido fluorídrico é largamente usado na indústria e no ambiente doméstico. Lesões graves podem ocorrer depois de contato com soluções altamente concentradas levando a necrose tecidual e destruição óssea. O tratamento específico é baseado na neutralização dos íons de flúor com soluções de cálcio ou magnésio.

**RELATO DE CASO:**

Homem de 41 anos foi atendido na sala de urgência 35 minutos depois de contato da pele com ácido fluorídrico a 70%, apresentando lesões esbranquiçadas e edemaciadas nos dedos médio e quarto da mão direita com dor intensa que iniciou logo após o contato. Pomada de gluconato de cálcio a 2,5% foi aplicada. Depois de 24 horas, o paciente continuava com dor mais intensa e as lesões haviam piorado. Considerando a concentração da solução, o início precoce da dor intensa, as características das lesões e a impossibilidade de administrar gluconato de cálcio no subcutâneo devido ao local da lesão, foi inserido cateter na artéria radial para infusão de gluconato de cálcio a 2% com bomba de infusão por 36 horas até melhora da dor. Nenhum efeito adverso foi observado durante o procedimento. Dez dias depois as lesões encontravam-se estáveis, sem alterações dos ossos vistas nos raios-X. Seis meses depois houve recuperação completa.

**CONCLUSÃO:**

Gluconato de cálcio intra-arterial pode ser considerado em queimaduras digitais por ácido fluorídrico. Recuperação completa dos dedos acometidos pode ser obtida com essa técnica mesmo que iniciada 24 horas após a exposição. Porém, ensaios clínicos controlados são necessários para confirmar a efetividade e a segurança desta intervenção.

## INTRODUCTION

Hydrofluoric acid (HF) is widely used in many industrial fields, including chemicals, fertilizers, pesticides, plastics, dyes, leather tanning, electrical sets and semiconductor manufacture. It is also used for domestic purposes like marble, brick and stone cleaning, rust removal and glass etching.^1^^,^^2^

Pain and erythematous lesions can take more than 24 hours to appear when the HF concentration is less than 20%. The symptoms may be delayed by eight hours at intermediate HF concentrations (20 to 50%). Acute intense pain always occurs at concentrations greater than 50%, even without immediate skin lesions.^1^^,^^2^ Accidents commonly affect fingers during applications of highly concentrated HF to stone, bricks, marble and glass surfaces. Severe local pain with possible destruction of the distal phalanx when finger burns are not adequately treated is frequently observed.^1^ The skin may appear normal initially, with only mild erythematous lesions, thus giving the false impression that the outcome would be favorable and erroneously downplaying the need for care.^1^ We describe a case of finger burns caused by highly concentrated HF that was treated with intra-arterial calcium gluconate 24 hours after the accident, with complete recovery.

## CASE REPORT

A 41-year-old Caucasian male was seen at the emergency department 35 minutes after skin contact with 70% HF solution in an occupational accident. He had been cleaning a slate floor around a pool using special gloves to do the job, but afterwards he touched the contaminated cork of the bottle without protection.

**Figure 1.**
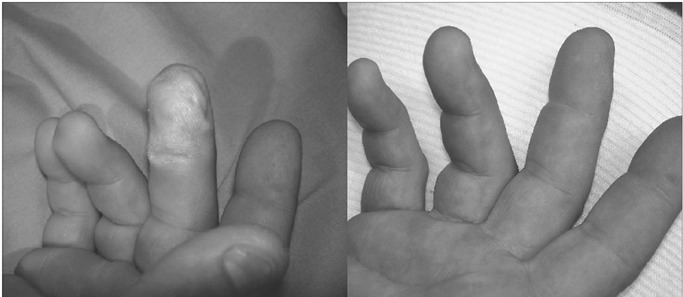
Lesion shown 24 hours after exposure and complete recovery seen six months later.

He presented lesions on the middle finger and part of the fourth finger of his right hand (Figure 1). He started feeling severe local pain immediately after contact (numerical rating scale, NRS = 8). He washed the lesions profusely with water for at least 15 minutes and then went to the emergency department. On arrival, the skin of the middle finger was already whitened and swollen, and he complained of severe local pain (NRS = 10). 2.5% calcium gluconate ointment was applied, and he was asked to continue to use it every two hours for the next 24 hours.

Twenty-four hours later, the patient was still in severe pain (NRS = 10) and the lesions had worsened. Serum calcium was 9.5 mg/dl. A bolus of 10% calcium gluconate (10 ml) was administered intravenously without any improvement of the pain. Considering the high concentration of the HF solution, the early start and maintenance of severe pain 24 hours after the accident, the whitened and swollen lesion suggesting progression to necrosis and the impossibility of subcutaneous administration of calcium gluconate because of the lesion location, it was decided to administer the drug intra-arterially through catheterization of the radial artery at wrist level. A solution of 2% calcium gluconate in 5% dextrose was given by means of an infusion pump every four hours for 36 hours, until the pain subsided (NRS = 3).

No adverse effects were seen, and the catheter was withdrawn without local problems. Ten days later, the lesions were stable with no sign of necrosis. Radiography on the right hand showed no bone abnormalities. Six months later, the patient presented complete recovery from the lesions (*restitutio ad integrum*) (Figure 1).

## DISCUSSION

HF is a fairly weak acid compared with sulfuric (H_2_SO_4_), hydrochloric (HCl) and nitric (HNO_3_) acids. Due to its very low dissociation constant (1000 times lower than HCl), HF presents rather weak release of hydrogen ions. The toxic mechanism relates mostly to its non-dissociated and uncharged chemical form, which can penetrate through the skin and subcutaneous tissues by means of non-ionic diffusion, targeting calcium-rich tissues like bones.^2^

After dissociation, the strong electronegativity of the fluoride ion allows it to bind tightly to any endogenous cation, particularly calcium (Ca) and magnesium (Mg), to produce insoluble salts.^2^ Soluble salts can also be formed with other cations that dissociate rapidly, thereby releasing fluoride ions again and leading to further tissue destruction. This toxic action can produce hypocalcemia, hypomagnesemia, cell necrosis, bone decalcification and destruction and cell dehydration with release of potassium ions (hyperkalemia).^2^ Differently from strong acids, it can take several days for HF to complete the process of tissue destruction and neutralization.

As acid penetrates, cell necrosis occurs and the skin can become whitened, eventually developing vesicles and progressing to bone destruction. Disproportionate pain in relation to the apparently benign skin lesion is quite typical of HF burns, and serves to alert to the need for aggressive treatment and careful follow-up.^1^

The typical presentation of more than 50% of HF lesions is whitened tissue surrounded by erythema, accompanied by severe pain.^3^ Such accidents should always be investigated for systemic effects, independently of the extent of the lesions.^3^

Topical therapy with application of 2.5% calcium gluconate gel can be attempted in mild or moderate cases.^4^ For finger burns, the ointment must be applied in a latex glove.

Intradermal or subcutaneous application of 10% calcium gluconate solution (never calcium chloride), around and into the affected area, can be of help in cases of lesions in which the subcutaneous tissue is loose enough to support gluconate deposits without interfering with normal blood circulation.^3^^,^^5^ Obviously, this cannot be used for finger lesions.

Intravenous calcium gluconate or magnesium sulfate must be given when serum calcium and magnesium imbalance is detected. Regional intravenous infusion of calcium can be used for finger lesions using a Bier block technique.^6^

Intra-arterial perfusion of calcium gluconate is a good alternative in cases of swollen and painful injured fingers. The advantages that can be listed for its use include rapid pain relief and delivery of a greater amount of calcium, with better distribution to tissues because of calcium induction of vasodilatation. Adverse effects and complications are rare and, for moderate to severe burns of fingers and hands (generally with HF concentrations > 10%), intra-arterial calcium infusion is thought to be more effective than local therapy.^3^^,^^5^

## CONCLUSION

Intra-arterial calcium gluconate might be considered for finger burns caused by highly concentrated HF, when topical treatment is considered useless, or when intradermal and subcutaneous calcium injections cannot be performed. Complete recovery of wounded fingers can be achieved with this technique, even if it is started 24 hours after the exposure, as seen in this report. Controlled clinical trials must be carried out to assess the effectiveness and safety of this intervention.
